# Identification of Variants Associated With Rare Hematological Disorder Erythrocytosis Using Targeted Next-Generation Sequencing Analysis

**DOI:** 10.3389/fgene.2021.689868

**Published:** 2021-07-19

**Authors:** Aleša Kristan, Tadej Pajič, Aleš Maver, Tadeja Režen, Tanja Kunej, Rok Količ, Andrej Vuga, Martina Fink, Špela Žula, Helena Podgornik, Saša Anžej Doma, Irena Preložnik Zupan, Damjana Rozman, Nataša Debeljak

**Affiliations:** ^1^Medical Centre for Molecular Biology, Institute of Biochemistry and Molecular Genetics, Faculty of Medicine, University of Ljubljana, Ljubljana, Slovenia; ^2^Department of Hematology, University Medical Centre Ljubljana, Ljubljana, Slovenia; ^3^Clinical Institute of Genomic Medicine, University Medical Centre Ljubljana, Ljubljana, Slovenia; ^4^Clinical Biochemistry, Faculty of Medicine, University of Maribor, Maribor, Slovenia; ^5^Centre for Functional Genomics and Bio-Chips, Institute of Biochemistry and Molecular Genetics, Faculty of Medicine, University of Ljubljana, Ljubljana, Slovenia; ^6^Department of Animal Science, Biotechnical Faculty, University of Ljubljana, Ljubljana, Slovenia; ^7^Kemomed Research and Development, Kemomed Ltd., Ljubljana, Slovenia; ^8^Clinical Biochemistry, Faculty of Pharmacy, University of Ljubljana, Ljubljana, Slovenia; ^9^Department of Internal Medicine, Faculty of Medicine, University of Ljubljana, Ljubljana, Slovenia

**Keywords:** NGS – next-generation sequencing, erythrocytosis, targeted panel sequencing, iron metabolism, diagnostics, rare disease (RD)

## Abstract

An erythrocytosis is present when the red blood cell mass is increased, demonstrated as elevated hemoglobin and hematocrit in the laboratory evaluation. Congenital predispositions for erythrocytosis are rare, with germline variants in several genes involved in oxygen sensing (*VHL*, *EGLN1*, and *EPAS1*), signaling for hematopoietic cell maturation (*EPOR* and *EPO*), and oxygen transfer (*HBB*, *HBA1*, *HBA2*, and *BPGM*) that were already associated with the eight congenital types (ECYT1–8). Screening for variants in known congenital erythrocytosis genes with classical sequencing approach gives a correct diagnosis for only up to one-third of the patients. The genetic background of erythrocytosis is more heterogeneous, and additional genes involved in erythropoiesis and iron metabolism could have a putative effect on the development of erythrocytosis. This study aimed to detect variants in patients with yet unexplained erythrocytosis using the next-generation sequencing (NGS) approach, targeting genes associated with erythrocytosis and increased iron uptake and implementing the diagnostics of congenital erythrocytosis in Slovenia. Selected 25 patients with high hemoglobin, high hematocrit, and no acquired causes were screened for variants in the 39 candidate genes. We identified one pathogenic variant in *EPAS1* gene and three novel variants with yet unknown significance in genes *EPAS1*, *JAK2*, and *SH2B3.* Interestingly, a high proportion of patients were heterozygous carriers for two variants in *HFE* gene, otherwise pathogenic for the condition of iron overload. The association between the *HFE* variants and the development of erythrocytosis is not clearly understood. With a targeted NGS approach, we determined an actual genetic cause for the erythrocytosis in one patient and contributed to better management of the disease for the patient and his family. The effect of variants of unknown significance on the enhanced production of red blood cells needs to be further explored with functional analysis. This study is of great significance for the improvement of diagnosis of Slovenian patients with unexplained erythrocytosis and future research on the etiology of this rare hematological disorder.

## Introduction

Erythrocytes or red blood cells are the most abundant cells in the blood with the main function of tissue oxygen delivery (Lee and Percy, 2011). An increase in red blood cell mass (RCM) for more than 125% of predicted for age and sex is defined as absolute erythrocytosis. Increased RCM leads to raised blood viscosity and manifests as elevated hemoglobin (Hb) and hematocrit (Ht). Different criteria of Hb and Ht have been published for the diagnosis of erythrocytosis, with the consensus that Hb > 185 g/L and Ht > 0.52 in men and Hb > 165 g/L and Ht > 0.48 in women in at least two separate blood counts at 2 months apart requires further investigations (Patnaik and Tefferi, 2009; Keohane et al., 2013; McMullin, 2014, 2016; Bento, 2018). Clinical signs are associated with increased blood viscosity and include non-specific signs like dizziness, itching, facial plethora, redness of the hands, pulmonary hypertension, and, in some patients, serious complications like thromboembolic events and death (Lee and Arcasoy, 2015; McMullin et al., 2019a,b).

**FIGURE 1:**
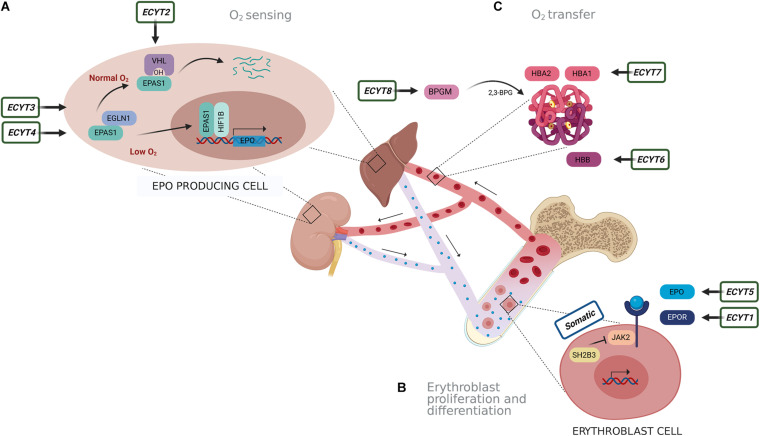
Schematic presentation of genes responsible for eight types of congenital erythrocytosis (ECYT1–8) and somatic erythrocytosis. Genes are involved in pathways of **(A)** oxygen sensing, **(B)** signaling for proliferation and differentiation of erythroblasts and **(C)** oxygen transfer. **(A)** Oxygen is detected in the liver and kidney. In the normal oxygen conditions, hypoxia-inducible transcription factor (HIF) (the main isoform associated with the erythropoiesis is EPAS1) is hydroxylated by prolyl hydroxylase EGLN1, following binding of *VHL*. This results in ubiquitination and degradation of HIF. Under hypoxic conditions (e.g., 1% O_2_), oxygen availability is limited; thus, hydroxylation is diminished. This results in the stabilization of HIF that is transported to the nucleus where it forms a dimer complex with its beta subunit HIF1B (officially termed ARNT) and acts as transcription factor on numerous target genes, including *EPO*. **(B)** EPO is transported to the bone marrow, where it binds with its receptor EPOR on the erythroblast cells. This subsequently activates JAK2 signaling cascade for proliferation and differentiation of erythroblasts. SH2B3 is an inhibitor of JAK2 kinase. **(C)** Hemoglobin alpha (HBA1 and HBA2) and beta (HBB) are important binding proteins for oxygen and are involved in oxygen transfer. The enzyme BPGM forms an allosteric effector 2,3-biphosphoglycerate (2,3-BPG) for regulation of hemoglobin oxygen affinity.

Several factors could lead to increased RCM. The cause of erythrocytosis could be congenital or acquired and can be further divided into primary and secondary. An erythrocytosis is classified as primary, when there is an intrinsic defect in the erythroid progenitor cells, and secondary when the defect outside the erythroid compartment is driving the bone marrow to produce more red blood cells (McMullin, 2016; Bento, 2018). The most common reasons for erythrocytosis are acquired, due to somatic variants or various extrinsic factors that lead to reduced oxygen supply and thus stimulation of erythropoiesis, such as chronic pulmonary, cardiac, renal, hepatic diseases, erythropoietin (EPO)-secreting tumors, high-altitude living, smoking, sleep apnea, recombinant EPO, and androgen administration (McMullin, 2008; Lee and Percy, 2011; Bento, 2018). Genetic defects in several pathways and mechanisms that regulate erythropoiesis lead to erythrocytosis, including oxygen-sensing pathway and regulation of EPO transcription, EPO signal transduction mediated *via* EPOR-JAK2 signaling cascade, and regulation of hemoglobin-oxygen affinity (Gaspersic et al., 2020). Primary acquired erythrocytosis, i.e., somatic erythrocytosis (OMIM ID: 133100), is the consequence of somatic genetic variants in Janus kinase 2 (*JAK2*) gene and SH2B adaptor protein 3 (*SH2B3*) gene, which lead to constant activation of EPO signaling pathway (McMullin and Cario, 2016; Maslah et al., 2017; Bento, 2018). Somatic variant p.Val617Phe and variants in exon 12 of the *JAK2* gene are the reason for the development of somatic erythrocytosis also termed polycythemia vera (PV) (OMIM ID: 263300) in 98% of erythrocytosis cases (Baxter et al., 2005; Scott et al., 2007; Bento, 2018). Cases with congenital erythrocytosis due to germline variants are much rarer. Based on the genetic origin, there are eight types of congenital (familiar) erythrocytosis, namely, ECYT1–8. Heterozygous variants in the EPO receptor gene (*EPOR*) are responsible for the development of ECYT1 (OMIM ID: 133100). The mechanism underlying ECYT2–5 (OMIM IDs: 263400, 609820, 611783, and 617907) is impairment of genes von Hippel–Lindau tumor suppressor (*VHL*), egl-9 family hypoxia-inducible factor 1 (*EGLN1*), endothelial PAS domain protein 1 (*EPAS1*), and *EPO* involved in the oxygen sensing. ECYT6–8 (OMIM IDs: 617980, 617981, and 222800) are developed due to variants in hemoglobin genes *HBB*, *HBA1*, and *HBA2* and biphosphoglycerate mutase (*BPGM*), which lead to increased oxygen affinity of hemoglobin (Figure 1; McMullin, 2016; Bento, 2018). Except for VHL and BPGM-associated erythrocytosis, all other familial types have shown an autosomal-dominant type of inheritance (Amberger et al., 2019). Besides the above-mentioned genes, also other genes are included in the pathways of red blood cell production but were not yet associated with the clinical outcome of erythrocytosis (Gaspersic et al., 2020).

It is increasingly evident that the same genes and pathways could be involved in the pathology of different diseases. Hereditary hemochromatosis (HH) is a disorder of systemic iron overload, due to the deficiency in iron regulatory hormone hepcidin. Variants in genes encoding HH protein (HFE), hemojuvelin (HJV), hepcidin (HAMP), transferrin receptor protein 2 (TFR2), and ferroportin (SLC40A1) could lead to loss of hepcidin transcription or activity and development of five HH types. The most common causes for HH are homozygous or compound heterozygous defects (p.Cys282Tyr, p.His63Asp, and p.Ser65Cys) in gene *HFE*, resulting in autosomal-recessive HH type 1 (Lanktree et al., 2017; Brissot et al., 2018). Recently, several research groups found *HFE* variants at higher frequencies among patients with unexplained erythrocytosis, i.e., idiopathic erythrocytosis (IE), compared with the general population (Biagetti et al., 2018; Burlet et al., 2019; Gurnari et al., 2019). Similarly, patients with the *HFE* variants had significantly higher Hb and Ht values than normal (Barton et al., 2000; Asif et al., 2019). It was suggested that *HFE* mutations could induce erythropoiesis through higher iron bioavailability, since iron is dedicated to the synthesis of Hb (Hentze et al., 2010; Biagetti et al., 2018; Burlet et al., 2019). Several gene panels of hemochromatosis and iron metabolism-associated genes have been developed (Badar et al., 2016; Faria et al., 2016; Ferbo et al., 2016; Lanktree et al., 2017).

In the past, most of the laboratories and diagnostic centers included only genes for ECYT1–8 in their routine laboratory evaluation for congenital erythrocytosis, using Sanger sequencing as a predominant method (Gaspersic et al., 2020). However, the practice has been so far that only about 20–30% of patients received a proper diagnosis with screening for known variants associated with erythrocytosis. Therefore, the majority of patients remained idiopathic (Bento et al., 2013; Camps et al., 2016; Girodon et al., 2017; Bento, 2018). This implicates that several other genes and mechanisms must be involved in the disease development. In recent years, the next-generation sequencing (NGS) approach was introduced into diagnostics of patients with IE or suspected congenital erythrocytosis. The biggest advantage of NGS sequencing in rare disease diagnostics is that it allows a massive parallel sequencing of multiple genomic regions of interest at once (Fernandez-Marmiesse et al., 2018). Several researchers used whole-genome sequencing (WGS) to identify novel candidate genes and variants involved in the development of congenital erythrocytosis (Taylor et al., 2015; Lenglet et al., 2018). To overcome the drawbacks of WGS, like high cost and processing a huge amount of data, targeted NGS panels were established. Camps et al. (2016) developed the erythrocytosis gene panel of 21 candidate genes, which are involved in key disease-driven pathways or were identified in prior WGS projects (Taylor et al., 2015; Camps et al., 2016). Targeted NGS of erythrocytosis-associated genes was later also used by a French research group studying erythrocytosis (Girodon et al., 2017; Filser et al., 2021), and erythrocytosis gene panels are gradually introduced into the routine clinical practice (Gaspersic et al., 2020).

The diagnostic procedures for the evaluation of patients with abnormally high Hb and Ht had been insufficient in Slovenia so far (Mlakar, 2008). A previous genetic analysis of patients with signs of erythrocytosis included only the examination of the *JAK2* gene for variants causative for PV, with allele-specific PCR, high-resolution melting (HRM) analysis, and Sanger sequencing (Baxter et al., 2005; Ugo et al., 2010; Geay et al., 2020). A huge proportion of patients with excluded PV were undiagnosed and remained idiopathic. It was urgent to modernize the diagnostic algorithm for erythrocytosis and to extend the genetic analysis for other known and novel candidate genes associated with the development of erythrocytosis. We have recently developed a national diagnostic algorithm for erythrocytosis, differentiating patients with absolute erythrocytosis, in whom acquired causes and PV are excluded (Anzej Doma et al., 2021a). The precise selection of patients to the point where genetic testing is considered is important to improve the diagnostic yield.

In the previous study, we have described and verified the targeted NGS method, which we developed for the genetic characterization of IE (Kristan et al., 2021). In the same gene panel, we included for the first time genes associated with erythrocytosis and HH. This was an important improvement to the erythrocytosis gene panels already used in international clinical practice since erythropoiesis and iron metabolism pathways are clearly intertwined. In the present study, we used targeted NGS to diagnose a group of patients with IE, which were selected over a period of 8 years (Anzej Doma et al., 2021a). The targeted NGS approach, in combination with the national diagnostic algorithm, improved the diagnostic accuracy of the Slovenian patients with IE, as we provided new explanations for the development of erythrocytosis to the patients.

## Materials and Methods

### Patients

Adult patients with erythrocytosis of unknown cause were selected based on the diagnostic algorithm for erythrocytosis (Anzej Doma et al., 2021a) from individuals followed up at University Medical Centre Ljubljana (UMCLj) between 2011 and 2019. All patients were of Slovenian ethnic origin. A complete hemogram was performed for the patients, with additional relevant hematological parameters like ferritin values and saturation of transferrin. The inclusion criteria were (a) confirmed absolute erythrocytosis with hemoglobin >185 g/L for men and 165 g/L for women or hematocrit >0.52 for men and >0.48 for women twice over at least 2 months; (b) absence of pathogenic *JAK2* variants p.Val617Phe and exon 12 variants for PV; and (c) absence of any defined cause of secondary acquired erythrocytosis. Participants gave informed consent; the study was approved by the National Medical Ethics Committee, Ministry of Health of the Republic of Slovenia, approval no. 115/07/15 (0120-198/2015-4, 0120-287/2019-4). Patients were also reviewed for a family history of erythrocytosis. Several patients reported similar symptoms of erythrocytosis in families, and we were able to gather additional samples of other family members. Overall, 25 patients were selected for the genetic analysis with targeted NGS: 21 unrelated patients and two families, each with two participating members. Additionally, a family member without erythrocytosis from one of the families was included. DNA samples were extracted from granulocytes as described earlier (Kristan et al., 2021). We also included commercial reference DNA control NA12878, obtained from the Coriell Institute.

### Targeted Next-Generation Sequencing and Data Analysis

We used targeted NGS to analyze samples from selected patients, one family member without erythrocytosis, and a reference DNA control. Library preparation and enrichment of 39 genes involved in erythropoiesis and hemochromatosis development were performed as described previously (Kristan et al., 2021). For enrichment, sample libraries were combined into multiplex pools of 12 samples pooled by mass. Enriched libraries were sequenced on MiniSeq sequencer (Illumina, San Diego, CA, United States) in 2 × 150 cycles. After duplicates were removed, the alignment of reads to UCSC hg19 reference assembly was done using BWA algorithm (v0.6.3), and variant calling was done using GATK framework (v2.8). Only variants exceeding the quality score of 30.0 and depth of 5 were used for downstream analysis. Variant annotation was performed using ANNOVAR and snpEff algorithms, with pathogenicity predictions in dbNSFPv2 database. Reference gene models and transcript sequences are based on the RefSeq database. Variants with population frequency exceeding 1% in 1000 genomes and ESP6500, synonymous variants, intronic variants, and variants outside the clinical target were filtered out during analyses (Bergant et al., 2018). Separately, we also looked for clinically significant variants in *HFE* gene, which has a higher population frequency than 1%. The interpretation of sequence variants was based on ACMG/AMP standards and guidelines (Richards et al., 2015), modified in accordance with the ACGS recommendations (Ellard et al., 2019). Variants are classified as follows: class 1, benign variants; class 2, possibly benign variants; class 3, variants of uncertain significance, not enough evidence; class 4, possibly pathogenic variants; and class 5, pathogenic variants. Classification of variants is supported by the evidence categories (Richards et al., 2015). A list of *in silico* tools with deleterious predictions of variants was obtained from the Varsome database^1^ (Kopanos et al., 2019). A variant was predicted to have a deleterious effect on the gene or gene product (evidence category PP3), when the majority of computational predictors or the majority of meta-predictors (REVEL, METALR, METASVM, and CADD) supported a deleterious effect. The cutoff value on deleteriousness for CADD (Combined Annotation Dependent Depletion) score was set to >20 (Rentzsch et al., 2019). The frequencies of identified variants in the general population were gathered from the GnomAD browser, version v2.1.1^2^. We reviewed databases ClinVar (Landrum et al., 2018), LOVD (Fokkema et al., 2011), HGMD (Stenson et al., 2003), COSMIC (Tate et al., 2019), and CIViC (Griffith et al., 2017) to search for clinically relevant data of variants. Gene abbreviations are according to the official symbols in HUGO Gene Nomenclature Committee (Tweedie et al., 2021). The purpose of the reference DNA sample was to serve as a control for sequencing performance. For benchmarking the method sensitivity, reference DNA NA12878 was sequenced, and variant calls were compared against the high-confidence variant calls provided by the NIST Genome in a bottle consortium (v.3.3.2).

### Validation Using Sanger Sequencing

All identified pathogenic variants were further confirmed by Sanger sequencing (GATC Biotech, Konstanz, Germany). Sanger sequencing and prior PCR amplification were performed with custom-designed primers (IDT) and are available upon request.

## Results

### Sequencing Performance

**FIGURE 2:**
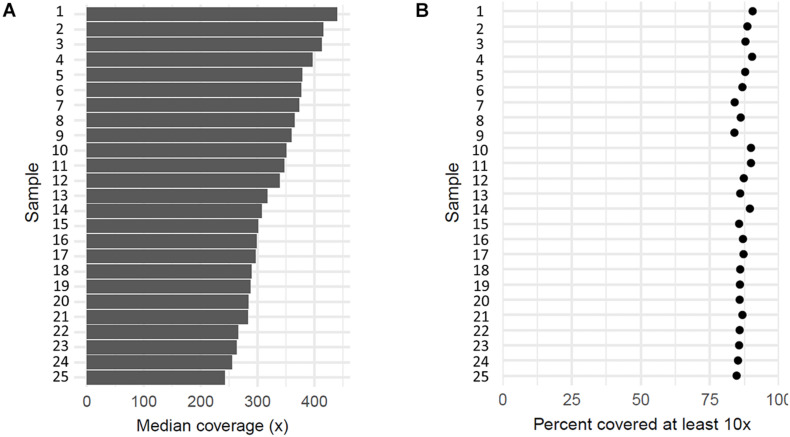
Coverage of 25 sequenced patient’s samples. **(A)** Each bar represents median coverage depth for an individual sample. **(B)** Each dot represents the percentage of target regions with coverage at least 10× for an individual sample.

All 27 samples, including patients, one individual without erythrocytosis, and a reference DNA control, were sequenced by the targeted NGS. On average, 87% of mapped reads were on target regions, which indicates a successful enrichment. Targeted sequenced bases had high quality, as over 92% of aligned bases were sequenced with the minimum quality of Q30. With sequencing, we reached the median coverage of 230× (Figure 2A). The capture design encompassed 377 regions, of which we attained an average coverage of at least 10× for 362 regions (96%) across the sequenced samples, indicating that the majority of captured regions were sequenced at sufficient coverage for sensitive variant detection (Figure 2B). Only 15 target regions (4.0%) had an average coverage lower than 10× across samples, probably due to the high guanine–cytosine (GC) content of these regions (Supplementary Table 1). We cross-referenced the regions with poor coverage against the published pathogenic or likely pathogenic variants in the ClinVar database and found that these regions do not contain any known erythrocytosis-associated clinically relevant variants.

Using our analysis approach, we detected 47 out of 51 variants within the targeted region of the NA12878 reference sample. Of these, three undetected variants were located within the known regions of poor coverage and were thus not considered in the sensitivity calculation. Apart from that, only a single variant was not detected in the sensitivity experiment due to the low coverage of the region in the control sample, corresponding to an estimated sensitivity of 97.9% for our sequencing approach.

### Identified Variants

**FIGURE 3:**
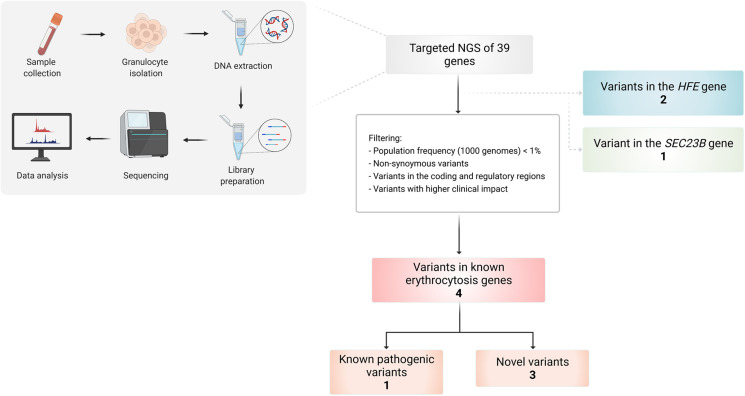
Workflow of the study and overview of the identified variants in selected patients using targeted NGS of 39 genes.

Several variants were identified using the erythrocytosis and hemochromatosis gene panel (Figure 3). Four variants were identified in the known erythrocytosis-causing genes *EPAS1*, *JAK2*, and *SH2B3* (Table 1). One variant in *EPAS1* gene had been already reported in the literature as causing erythrocytosis, while the remaining variants were not yet clinically associated with erythrocytosis. In addition, we also identified variants from the hemochromatosis set of genes, two *HFE* variants (Table 2) and one in the SEC23 homolog B (*SEC23B*) gene, which was found to be associated with a different condition. All the identified variants were located in the coding regions of the analyzed genes.

**TABLE 1:** Known and novel variants detected in the known erythrocytosis genes.

Genomic location on hg19	Gene	Coding DNA change (RefSeq transcript)	Protein change	RS number	Allele frequency (GnomAD)	*In silico* tools with deleterious predictions^1^ (CADD value)	Patient information^2^	Genotype	Classification^3^ (evidence categories)
Chr.2:46607420	*EPAS1*	c.1609G>A (NM_001430.5)	p.(Gly537Arg)	rs137853036	NA	18/22: BayesDel addAF, BayesDel noAF, DANN, DEOGEN2, EIGEN, EIGEN PC, FATHMM-MKL, FATHMM-XF, LIST-S2, LRT, MVP, MutPred, Mutation assessor, MutationTaster, PROVEAN, PrimateAI, SIFT, SIFT4G; meta predictors: CADD (29.7)	Female; age, 21 years; Hb, 197 g/L; Ht, 0,59; RBC 6,35 × 10^12^/L; platelets, 184 × 10^9^/L; EPO, 73.4 IU/L; smoking; several thromboembolic events; pulmonary hypertension; erythrocytosis since childhood; phlebotomy; anticoagulation therapy	Het	Pathogenic (PS1, PS3, PS4, PM1, PM2, PM5, PP1_STR, PP4)
Chr.9:5072617	*JAK2*	c.1767C>A (NM_004972.4)	p.(Asn589Lys)	rs1362123436	0.000004	12/22: DANN, DEOGEN2, FATHMM, FATHMM-MKL, LIST-S2, LRT, Mutation assessor, MutationTaster, PROVEAN, PrimateAI, SIFT, SIFT4G; meta predictors: CADD (23.6)	Male; age, 62 years; Hb, 221 g/L; Ht, 0.65; RBC, 6.36 × 10^12^; EPO 11.4 IU/L; platelets, 211 × 10^9^/L; smoking; elevated blood pressure and heart rate; phlebotomy; positive family history, son also affected but without variant	Het	VUS (PM2)
Chr. 2:46608809	*EPAS1*	c.2120A>C (NM_001430.4)	p.(Lys707Thr)	rs950180639	NA	12/21: BayesDel addAF, BayesDel noAF, DANN, DEOGEN2, EIGEN PC, FATHMM-MKL, FATHMM-XF, LIST-S2, Mutation assessor, MutationTaster, SIFT, SIFT4G; meta predictors: CADD (26)	Male; age, 54 years; Hb, 182 g/L; Ht, 0.53; RBC, 6.24 × 10^12^; EPO 6.1 IU/L; platelets, 271 × 10^9^/L; smoking; mild sleep apnea; asthma; Hashimoto’s thyroiditis, phlebotomy; possible positive family history, brother also similar symptoms	Het	VUS (PM2)
Chr. 12:111884812	*SH2B3*	c.901G>A (NM_005475.3)	p.(Glu301Lys)	rs374278232	0.00004	15/21: BayesDel noAF, DANN, DEOGEN2, EIGEN, EIGEN PC, FATHMM-MKL, FATHMM-XF, LRT, MVP, MetaLR, MetaSVM, MutationTaster, PROVEAN, PrimateAI, SIFT, SIFT4G; meta predictors: MetaSVM, CADD (28.4)	Male; age, 57 years; Hb, 181 g/L; Ht, 0.53; RBC, 6.05 × 10^12^; EPO 10.1 IU/L; platelets, 226 × 10^9^/L; possible sleep apnea; aspirin; no positive family history	Het	VUS (PP3)

*VUS, variant of unknown significance; Het, heterozygous; Hb, hemoglobin; Ht, hematocrit; RBC, red blood cells; EPO, erythropoietin; NA, not available; hg19, genome assembly GRCh37/hg19.*

*^1^Number of pathogenicity predictions tools out of all indicated *in silico* tools with deleterious effect were collected from the Varsome database.*

*^2^The indicated hematological parameters (Hb, Ht, RBC, and platelets) and age were registered at the first examination; however, additional measurements have been made later, and Hb and Ht were elevated at least twice over > 2 months but are not reported here.*

*^3^Classification of variants based on ACMG/AMP standards.*

*Hb normal range: 130–170 g/L for male and 120–150 g/L for female; Ht normal range: 0.40–0.50 for male and 0.36–0.46 for female; RBC normal range: 4.5–5.5 × 10^12^/L for male and 3.8–4.8 × 10^12^/L for female; platelet normal range: 150–410 × 10^9^/L; EPO normal range: 3.3–16.6 IU/L; ferritin normal range: 20–300 μg/L for male and 10–120 μg/L for female; transferrin saturation normal range: 0.15–0.45.*

**TABLE 2:** Distribution of *HFE* variants among IE patients and number of variant carriers with elevated ferritin and transferrin saturation.

Genomic location on hg19	Coding DNA change (RefSeq transcript)	Protein change	RS number	Genotype	Number of patients (*N* = 25)	Proportion of patients (%)	Allele frequencies	Allele frequencies in Slovenian population^1^	Variant carriers with elevated ferritin	Variant carriers with elevated transferrin saturation
Chr. 6:26093141	c.845G>A (NM_000410.3)	p.(Cys282Tyr)	rs1800562	Het	4	0.16	0.08	0.036	1	2
Chr. 6:26091179	c.187C>G (NM_000410.3)	p.(His63Asp)	rs1799945	Het	6	0.24	0.12	0.128	0	1

*Het, heterozygous; hg19, genome assembly GRCh37/hg19.*

*^1^Allele frequencies were obtained from the 1,282 Slovenian blood donors tested for the *HFE* variants (Cukjati et al., 2007).*

*Ferritin normal range: 20–300 μg/L for male and 10–120 μg/L for female. Transferrin saturation normal range: 0.15–0.45.*

#### Known Pathogenic Variants

Heterozygous variant c.1609G>A in *EPAS1* gene is a pathogenic variant that represents an established cause of familial erythrocytosis type 4 (ECYT4, OMIM ID 611783). This variant has been previously reported as pathogenic in numerous unrelated individuals with erythrocytosis (Gale et al., 2008; Percy et al., 2008a; Perrotta et al., 2013; Liu et al., 2017; Oliveira et al., 2018). In addition, it has been reported to co-segregate with erythrocytosis in multiple affected family members (Gale et al., 2008; Percy et al., 2008a; Liu et al., 2017). The variant is also classified as pathogenic in the ClinVar, LOVD, and HGMD databases (ClinVar ID: 6469, LOVD ID: EPAS1_000002, HGMD ID: CM081583); and the deleterious effect on the protein was confirmed in functional studies (Gale et al., 2008; Furlow et al., 2009; Perrotta et al., 2013; Tarade et al., 2018). The clinical presentation of the patient was highly consistent with the disease.

#### Novel Variants in Known Candidate Genes

Three heterozygous variants c.2120A>C, c.1767C>A, and c.901G>A with unknown clinical impact were identified in known erythrocytosis-associated genes *EPAS1*, *JAK2*, and *SH2B3* genes, respectively.

Heterozygous variant c.901G>A in *SH2B3* gene has been already recorded in the HGMD database (ID: CM1614293). It was previously reported in one patient with IE without a family history of erythrocytosis (Camps et al., 2016).

On the contrary, heterozygous variant c.2120A>C in *EPAS1* gene has not yet been reported in association with human diseases in the literature and is not recorded among the clinically relevant variants in ClinVar, LOVD, and HGMD databases at the time of data analysis.

Similarly, heterozygous variant c.1767C>A in *JAK2* gene has not been yet recorded among the clinically relevant variants in ClinVar, HGMD, COSMIC, and CIViC databases. The variant in *JAK2* gene was not found in other affected or unaffected relatives, and the allele fraction [variant allele frequency (VAF)] was calculated to be 22%, which indicates its possible somatic origin.

We classified all three variants as variants with unknown significance (VUS) due to low population frequency in the GnomAD project or high pathogenicity. Variants in *EPAS1* and *JAK2* genes were observed at extremely low frequencies in the gnomAD project or were absent from the GnomAD controls. Variant *SH2B3* c.901G>A was predicted to have a deleterious effect on the protein as the majority of meta-predictors (REVEL, METASVM, and CADD) supported its deleteriousness.

#### Variants in the Hemochromatosis Genes

With the targeted NGS analysis, we identified two heterozygous variants p.(Cys282Tyr) and p.(His63Asp) in *HFE* gene. Out of 25 patients with IE, 10 patients were carriers for variant p.(Cys282Tyr) or variant p.(His63Asp). Comparing the observed VAFs with the allele frequencies in the Slovenian population (Cukjati et al., 2007), IE patients had a higher incidence of variant p.(Cys282Tyr). Only a few patients with identified heterozygous *HFE* variants had elevated ferritin and saturation of transferrin > 45%, which is indicative of iron overload (Table 2).

Additionally, we identified a carriership of a pathogenic missense c.40C>T (NM_006363.4) variant in *SEC23B* gene, associated with the congenital dyserythropoietic anemia, type 2 (CDAII; OMIM:224100) in two unrelated patients. The significance of this finding in the development of erythrocytosis is not clear and is less likely causative concerning a high frequency of heterozygous variant carriers in the population. This variant was therefore excluded from the further report and interpretation of variants.

## Discussion

With previously established targeted NGS of 39 erythrocytosis and hemochromatosis-associated genes, we identified high-confidence variants among patients with IE and discovered few potential disease-causing variants in patients who previously lacked a specific diagnosis. We were able to provide an actual genetic cause (variant p.Gly537Arg in *EPAS1* gene) for erythrocytosis in one patient, which was also validated by Sanger sequencing. This is the first patient in Slovenia with a conclusive diagnosis of congenital erythrocytosis type 4 (ECYT4). Relatives of this patient will be further included in the study and genetically tested, to provide proper prognosis and genetic counseling to the family. Three novel candidate variants in the genes known to be associated with erythrocytosis, i.e., *EPAS1*, *JAK2*, and *SH2B3*, were identified. Interestingly, another research group studying erythrocytosis identified the majority of variants in genes *EPAS1*, *SH2B3*, *JAK2*, and *EGLN1* (Girodon et al., 2021). Variant c.901G>A (NM_005475.2) in *SH2B3* gene was the only one already identified in the erythrocytosis patient without myeloproliferative neoplasm (MPN) (Camps et al., 2016). Nevertheless, further segregation analyses and functional studies are necessary to support and ascertain its pathogenic nature.

Among all identified variants, the variants located within or in the vicinity of the important regions for protein function will have a higher likelihood of causality. The identified pathogenic *EPAS1* variant c.1609G>A (p.Gly537Arg) is located six amino acids from the hydroxylation site p.Pro531, crucial for the oxygen-dependent regulation of EPAS1 stability (Kristan et al., 2019). Another amino acid change (c.1609G>T; p.Gly537Trp) (Percy et al., 2008b) (evidence category PM5) and the same amino acid change caused by a different base substitution (c.1609G>C; p.Gly537Arg) (Oliveira et al., 2018) (evidence category PS1) have been reported as pathogenic in patients with erythrocytosis. Furthermore, several amino acid substitutions c.1601C>G (p.Pro534Arg), c.1601C>T (p.Pro534Leu), c.1603A>G (p.Met535Val), c.1603A>T (p.Met535Leu), c.1604T>C (p.Met535Thr), c.1615G>A (p.Asp539Asn), c.1620C>A (p.Phe540Leu), and c.1631C>G (p.Pro544Arg) in the immediate vicinity of the identified variant have been reported in patients with erythrocytosis as pathogenic (Bento, 2018; Kristan et al., 2019), suggesting the critical importance of the identified region for the function of EPAS1 protein and severe effect on patient’s phenotype (Anzej Doma et al., 2021b). Some previous studies have shown that identified variant c.1609G>A (p.Gly537Arg), similar to other variants located C-terminal to p.Pro531, inhibits the binding of the EGLN1 hydroxylase to the EPAS1 protein. This results in impaired degradation and thus enhanced stabilization of EPAS1, which affects increased expression of target genes (Gale et al., 2008; Furlow et al., 2009; Perrotta et al., 2013; Tarade et al., 2018). Another identified variant in *EPAS1* gene, c.2120A>C (p.Lys707Thr), is located far downstream of the primary hydroxylation site, unlikely to have an effect on hydroxylation and degradation. However, the variant is located within the nuclear localization signal (NLS) at the C-terminus of the EPAS1 protein. Two basic NLS motifs were shown to be present in region 705–742 amino acids and are required for localization of transcription factor EPAS1 to the nucleus under hypoxia and normoxia. Furthermore, the introduced variant p.Lys709Thr partly impaired the nuclear accumulation of EPAS1 induced by hypoxia (Luo and Shibuya, 2001). This, together with the low frequency of variant among GnomAD controls, implies the potential effect of variant p.Lys707Thr on protein function and activity. Further functional studies are needed to provide further evidence of variant causality. No variants in the NLS region were previously associated with ECYT4, indicating that analysis of this region may be of importance in further diagnoses.

Gene *JAK2* codes for a non-receptor tyrosine kinase, in which its roles are to phosphorylate tyrosine residues in the EPO receptor and signal transducers and activators of transcription (STATs) and to activate the transcription of target genes for proliferation and differentiation of hematopoietic stem cells. The protein JAK2 has four major domains, with two Jak homology (JH) domains important for downstream signaling. The C-terminal JH1 domain (849–1124 aa) is a kinase domain responsible for phosphorylation, while structurally similar pseudo-kinase domain (JH2) (545–809 aa) lacks catalytic activity, and it is involved in auto-inhibition of JAK2 in the absence of ligand–receptor interaction (Gnanasambandan and Sayeski, 2011; Seif et al., 2017; UniProt Consortium., 2021). Variants in exons 12–15 were found in patients with various hematological disorders, and those exons (except for the exon 12) are located in the JH2 domain (Ma et al., 2009; Gnanasambandan and Sayeski, 2011). The most common somatic pathogenic variant in the exon 14, p.Val617Phe, which is responsible for the development of PV and other MPNs, allows the kinase to evade negative regulation and confer constitutive activation of JAK2 (Gnanasambandan and Sayeski, 2011). Identified variant p.(Asn589Lys) in the exon 13 of *JAK2* gene is also located in the auto-inhibitory domain JH2; therefore, the potential pathophysiological mechanism for enhanced erythrocytes production could be the same.

SH2B3 protein (also termed LNK) is a negative regulator of erythropoiesis, as it binds to the cytokines and JAK2 and inhibits downstream signaling pathways (Maslah et al., 2017). SH2B3 has three domains: a dimerization domain, central pleckstrin homology domain (PH), and a Src homology 2 (SH2) domain. The PH domain (194–307 aa) has a role in binding the adapter protein SH2B3 to the plasma membrane, which is important for early inhibitory action during cytokine signaling. A mutational hotspot in the SH2B3 protein is present in the PH domain, encompassing exons 2, 3, and 4. Identified *SH2B3* variant in our study was located in exon 4, at the C-terminus of the PH domain. The majority of the *SH2B3* variants found in patients with PV and also in patients with p.Val617Phe-negative erythrocytosis were located in the PH domain, with the amino acid residue 208 (exon 2) as a preferential mutational target site (Spolverini et al., 2013; McMullin and Cario, 2016; Maslah et al., 2017).

Somatic variants in *JAK2* and *SH2B3* genes are the cause for somatic erythrocytosis (OMIM ID: 133100), somatic myelofibrosis (OMIM ID: 254450), and thrombocythemia (OMIM ID: 614521, 187950) (McMullin and Cario, 2016; Amberger et al., 2019). In addition, germline variants in the *JAK2* gene are causative for thrombocythemia 3 (OMIM ID: 614521) (Mead et al., 2012; Amberger et al., 2019); and in several cases, germline *JAK2* variants, also in combination with other variants, were responsible for the activation of JAK2/STAT signaling in hereditary erythrocytosis or PV patients (Kapralova et al., 2014, 2016; Milosevic Feenstra et al., 2016; Wu et al., 2018). Heterozygous variant identified in the *JAK2* gene was present in only one affected family member and was observed in 22% of the sequence reads, as VAF was 0.22. The expected VAF for heterozygous germline variants is around 50%, with the distribution from 40 to 60%. For the somatic variants, VAF deviates from the germline and is usually lower (<40%), because the acquired variant is not present in all cells (Montgomery et al., 2018; Baer et al., 2019). This together indicates that JAK2 p.(Asn589Lys) could be of somatic origin; however, further testing of different type of sample (e.g., buccal swab) is required to confirm that. We already reported about two additional variants in the same individual with the JAK2 p.(Asn589Lys) variant, missense in the *EGLN1* gene and intronic in the *JAK2*, that showed segregation in the family (Kristan et al., 2021). In the present study, we used different filtration parameters for the selection of variants as in the previous study by Kristan et al. (2021); therefore, those two variants were filtered out. None of the two variants were directly involved in the development of erythrocytosis; however, the correlation between the variants, together with the novel, potential somatic variant in the *JAK2* gene, should be tested.

Besides known erythrocytosis genes, additional genes involved in the HIF and other erythropoiesis and iron metabolism pathways were included in the gene panel, in an attempt to discover novel candidate genes for the development of erythrocytosis. Unfortunately, we did not identify any novel disease-driver genes to be implicated in the erythropoiesis; however, we recognized a high incidence of heterozygous variants in *HFE* gene among the studied group of IE patients. This confirmed the observations of other researchers (Biagetti et al., 2018; Burlet et al., 2019; Gurnari et al., 2019). The difference of allele frequencies among our population of IE patients and the general population was larger for variant p.(Cys282Tyr). However, due to the small number of patients, this finding is of limited importance. Although homozygous variants p.(Cys282Tyr) have a high prevalence, the penetrance is low, as only 25–60% of patients develop clinical signs and only 55–82% of patients have increased serum ferritin level (Brissot et al., 2018). Low penetrance of causal *HFE* variants could indeed explain elevated ferritin values and saturation of transferrin in a small number of patients from our cohort. It would be useful to compare ferritin and saturation of transferrin values between larger cohorts of IE patients with *HFE* variants and IE *HFE*-wt patients, to see if there is any statistical significant difference. Biagetti et al. (2018) showed a higher frequency of elevated ferritin in mutated patients; however, the difference was not statistically significant due to a small group of patients (Biagetti et al., 2018). The pathophysiological mechanism between *HFE* variants and enhanced erythropoiesis is not clearly understood. In patients with high ferritin and transferrin values, this correlation could be explained by iron overload. However, this is not the case in patients with normal values; therefore, additional mechanisms must be involved. One possible explanation could be the linkage between the HIF pathway and iron metabolism. Schwartz et al. (2019) showed that low levels of hepatic iron regulator hepcidin lead to iron uptake and stabilization of EPAS1, through decreased prolyl-hydroxylase EGLN1 activity, as iron is an important co-factor for hydroxylation. Stabilized duodenal EPAS1 than activates target genes which regulate iron efflux (Schwartz et al., 2019). However, Schwartz et al. (2019) focused on the regulation of EPAS1 in duodenal enterocytes, and it would be interesting to study this mechanism also in other cell types and potentially find a connection with increased erythropoiesis.

With our sequencing approach, we achieved a high accuracy of sequenced bases, and high percentage of targeted regions had sufficient depth of coverage for germline variant calling. The sensitivity of our approach was high, as we accurately detected approximately 98% of variants calls in the reference NA12878 sample. Nevertheless, some limitations of the method were recognized and should be taken into consideration for future applications. The biggest limitation was insufficient coverage of some target regions. The majority of the target regions with poor coverage were seen in the first coding exons, as those regions are GC rich. Inadequate coverage in the first exon regions with high GC content is a common problem in the NGS approach, and usually other methods are applied to sufficiently sequence those regions (Chen et al., 2013). Another shortcoming of the applied method was the detection of variants in *HBA1* and *HBA2* genes, which was also observed by other researchers (Girodon et al., 2017; Filser et al., 2021). NGS, particularly read alignment for these two genes, is challenging, due to high sequence similarity. For future analysis of genes with high homology, a classical sequencing approach is suggested. One limitation of the study was also a low number of studied patients, but this was expected, as congenital erythrocytosis is a rare disorder. We identified only a few pathogenic variants and VUS with a likely causative mechanism in 16% (four out of 25) of patients with high Hb and Ht included in the study. This percentage is similar to the proportion of identified variants observed by other researchers, despite differences in the gene panel (Girodon et al., 2021). This indicates that the number of genes and gene regions included in the targeted panel must be extended. Also, we should consider the possibility that we missed larger structural variants, for instance, larger deletions, insertions, translocations, or copy number variations, that are harder to detect with limited targeted NGS or Sanger sequencing. To bypass this limitation, whole-exome sequencing (WES) or WGS needs to be applied in the future, especially the long-read sequencing approach for more sensitive and specific identification of larger variants.

## Conclusion

Through the study, we examined the patients with suspected congenital erythrocytosis for the first time with the established targeted NGS in the Slovenian clinical setting. As far as we know, this is the first established erythrocytosis gene panel for NGS, which includes genes associated with enhanced erythropoiesis and iron uptake. We showed that targeted NGS was indeed useful to explore variants in cases with suspected congenital erythrocytosis; however, in only approximately 15% of the patients, clinically interesting variants were identified. We identified ECYT4 with a known pathogenic variant in *EPAS1* gene and provided a diagnosis for one patient enabling proper decision regarding prognosis, genetic counseling, and treatment. Three VUS were identified in the known erythrocytosis genes, i.e., *EPAS1*, *JAK2*, and *SH2B3*. Further functional studies are necessary in order to elucidate the mechanism of action and explore the effect of variants on the development of erythrocytosis. Additionally, the germline or somatic origin should be clarified for the variants in *JAK2* and *SH2B3*, as those two genes are commonly associated with the somatic type of erythrocytosis.

In several patients, we detected two heterozygous variants in *HFE* gene, which confirmed the observations of other researchers. However, for the comprehensive study of the involvement of the *HFE* variants in erythropoiesis, we need to perform additional research on a larger group of patients.

For the remaining patients with no identified variants, broader approaches like WGS and WES should be applied, to detect the possible genetic cause of congenital erythrocytosis.

## Data Availability Statement

The datasets presented in this article are not readily available because due to concerns regarding participant/patient anonymity. Requests to access the datasets should be directed to the corresponding author.

## Ethics Statement

The studies involving human participants were reviewed and approved by the Slovenian National Medical Ethics Committee, Ministry of Health of the Republic of Slovenia. The patients/participants provided their written informed consent to participate in this study.

## Author Contributions

AK, ND, and IPZ contributed to conception and design of the study. IPZ, SAD, MF, TP, and HP revised clinical data. AK, TR, TK, RK, AV, and ND contributed with design of targeted NGS approach. AK, TP, AM, and ŠŽ performed the NGS analysis. AK wrote the first draft of the manuscript. TP, AM, and ND wrote sections of the manuscript. All authors contributed to manuscript revision, read, and approved the submitted version.

## Conflict of Interest

RK and AV are employees of the Kemomed Ltd., Kemomed Research and Development. The remaining authors declare that the research was conducted in the absence of any commercial or financial relationships that could be construed as a potential conflict of interest.

## Footnotes

1 https://varsome.com/

2 https://gnomad.broadinstitute.org/
