# Supplementary material for: Identification of Variants Associated With Rare Hematological Disorder Erythrocytosis Using Targeted Next-Generation Sequencing Analysis
Source: Front Genet. 2021 Jul 19;12:689868. doi: 10.3389/fgene.2021.689868 (PMC8327209; doi:10.3389/fgene.2021.689868)
Supplement: Supplementary file 1 [file Table_1.DOCX]

| *Target region (hg19)* | *Gene* | *Gene region* | *Transcript (refSeq ID)* |
| --- | --- | --- | --- |
| chr19:41305047-41305169 | *EGLN2* | exon 1 | NM_080732.4 |
| chr12:111855948-111856683 | *SH2B3* | exon 2 | NM_005475.3 |
| chr19:46800332-46800361 | *HIF3A* | exon 1 | NM_152795.4 |
| chr3:133465283-133465329 | *TF* | exon 1 | NM_001063.4 |
| chr15:43029209-43029302 | *CDAN1* | exon 1 | NM_138477.4 |
| chr14:62162521-62162560 | *HIF1A* | exon 1 | NM_001530.4 |
| chr19:41305333-41305545 | *EGLN2* | exon 1 | NM_053046.4, NM_080732.4 |
| chr19:11491730-11491887 | *EPOR* | exon 5 | NM_000121.4 |
| chr2:46525049-46525078 | *EPAS1* | exon 1 | NM_001430.5 |
| chrX:44732796-44732960 | *KDM6A* | exon 1 | NM_021140.3 |
| chr19:46811918-46812034 | *HIF3A* | exon 4 | NM_022462.4 |
| chr19:11492366-11492527 | *EPOR* | exon 4 | NM_000121.4 |
| chr19:46807134-46807347 | *HIF3A* | exon 2 | NM_022462.4 |
| chr7:100230850-100230967 | *TFR2* | exon 5 | NM_003227.4 |
| chr19:41306476-41307322 | *EGLN2* | exon 2 | NM_080732.4, NM_053046.4 |

**Supplementary table 1: Target regions with an average coverage lower than 10x across samples**
